# Artificial intelligence for infection surveillance, risk stratification, and antimicrobial decision support in acute-care hospitals: a scoping review

**DOI:** 10.3389/frai.2026.1859410

**Published:** 2026-06-12

**Authors:** Mohammad Hussein Mustafa, Mohammad S. Abu-Mahfouz, Samer Abdelmuhsen Saleh, Wesam Taher Almagharbeh, Sommanah Mohammed Alturaiki, Rabie Adel El Arab

**Affiliations:** 1 Dr. Sulaiman Alhabib Medical Group, Riyadh, Saudi Arabia; 2 Almoosa College of Health Sciences, Al Ahsa, Saudi Arabia; 3 Medical and Surgical Nursing Department, Faculty of Nursing, University of Tabuk, Tabuk, Saudi Arabia

**Keywords:** antimicrobial stewardship, artificial intelligence, cross infection, infection control, ventilator-associated pneumonia, surgical wound infection, urinary tract infections

## Abstract

**Background:**

Artificial intelligence (AI) has increasingly been proposed to strengthen infection surveillance, early risk stratification, antimicrobial decision support, and selected workflow functions in acute-care hospitals. However, the literature remains clinically heterogeneous, methodologically uneven, and conceptually fragmented, with technical performance often interprested too readily as evidence of clinical effectiveness. This scoping review aimed to map and synthesise the empirical literature on AI applications for infection surveillance and related hospital applications, while explicitly distinguishing technical performance from clinical utility, implementation relevance, and patient benefit.

**Methods:**

We conducted a scoping review in accordance with PRISMA 2020 and PRISMA-ScR guidance. CINAHL, Cochrane Library, Embase, PubMed, Scopus, and Web of Science were searched for English-language empirical studies. Eligible studies examined AI applications relevant to infection surveillance, risk prediction, detection, antimicrobial decision support, or workflow-relevant hospital functions in acute-care settings. Findings were synthesised narratively by application domain and translational stage.

**Results:**

Database searches yielded 884 records; 628 unique records underwent title and abstract screening, 180 full texts were assessed, and 39 studies were included. The literature was dominated by retrospective model-development and validation studies; no randomised trials or robust comparative evaluations under routine clinical conditions were identified. Evidence clustered around surgical-site infection, urinary-tract-infection-related outcomes, ventilator-associated pneumonia, bacteraemia, sepsis, resistant organisms, and infection-related mortality. Across these domains, AI models generally showed moderate-to-high discriminatory performance, particularly for surgical-site infection surveillance and prediction. A smaller body of evidence suggested potential operational value in antimicrobial prescribing support, early risk stratification, real-time bacteraemia prediction, and reduction of manual surveillance workload. However, implementation evidence was sparse and heterogeneous, with limited assessment of usability, adoption, trust, workflow redesign, sustained real-world performance, or patient-level benefit.

**Conclusion:**

AI shows substantial promise as an adjunct to infection surveillance and selected hospital infection-management tasks, but the current evidence base is considerably stronger for technical accuracy than for clinical effectiveness, implementation success, or patient benefit. Stronger prospective, externally validated, and implementation-oriented studies are needed before firmer claims can be justified.

## Introduction

Healthcare-associated infections (HAIs) are infections acquired during medical or surgical care in healthcare settings and were neither present nor incubating at the time of admission; they may also become clinically apparent after discharge (Asokan et al., 2026). HAIs remain major contributors to morbidity, mortality, prolonged hospital stay, and adverse clinical outcomes among hospitalised patients (Canadian Nosocomial Infection Surveillance Program1, 2024). Beyond their clinical burden, HAIs impose substantial health-system costs by prolonging hospitalisation, increasing resource utilisation, and requiring additional diagnostic, therapeutic, and supportive care (Orlando et al., 2025; Moradi et al., 2024; Asegu et al., 2024). Estimates suggest that HAIs account for more than 2.5 million cases annually in the EU/EEA and affect approximately one in ten or more hospitalised patients globally, with prevalence varying substantially by region and healthcare setting (Cassini et al., 2016; Raoofi et al., 2023). Despite substantial advances in infection prevention and control, antimicrobial stewardship, and surveillance, the burden of HAIs in hospital settings remains considerable, reflecting the multifactorial nature of these infections and the continuing need for sustained, system-level prevention and management strategies (Raoofi et al., 2023; Sartelli et al., 2024; Liu et al., 2023). Active surveillance is widely regarded as a cornerstone of infection prevention and control, providing the data needed to detect HAIs, monitor trends, guide targeted interventions, and evaluate prevention efforts (Shenoy and Branch-Elliman, 2023; Lukasewicz Ferreira et al., 2024). However, conventional surveillance approaches are often limited by the volume and complexity of infection-related data, the labour-intensive nature of manual chart review, variability in case ascertainment, and the difficulty of capturing adherence to infection-control practices in real time (Shenoy and Branch-Elliman, 2023; Zhang et al., 2023; van Mourik et al., 2021). Manual surveillance is also resource-intensive and time-consuming, requiring specialist infection-prevention expertise to interpret clinical data and apply standardised case definitions (Sartelli et al., 2024; Shenoy and Branch-Elliman, 2023). Consequently, automated and semi-automated surveillance has increasingly been regarded as an important means of strengthening HAI surveillance, enabling timelier detection and supporting earlier, more targeted infection-prevention and control responses (Shenoy and Branch-Elliman, 2023; Li et al., 2024; Verberk et al., 2022). Although earlier technological and data-infrastructure limitations constrained automation, recent advances in electronic health records, machine learning, and artificial intelligence (AI) have increased the feasibility of automated, data-driven HAI surveillance (Branch-Elliman et al., 2023; Radaelli et al., 2024). AI is particularly attractive in data-intensive surveillance contexts because it may support the rapid analysis of large, multidimensional datasets, augment rather than replace clinical judgement, improve consistency in case detection, and reduce reliance on labour-intensive manual review (Lambert et al., 2023; Chen et al., 2024; El Arab et al., 2025). Within infectious-disease and hospital infection contexts, AI has been explored across several domains, including pathogen surveillance, laboratory- and imaging-based detection, antimicrobial-resistance prediction, microbiome- and metagenomic investigation, and clinical decision support (El Arab et al., 2025; Abbara et al., 2025; Zhang et al., 2024; Li et al., 2024). These applications are not, however, methodologically or conceptually equivalent. In this review, they are therefore differentiated analytically into three domains: surveillance and risk prediction, infection-management support, and workflow-facing operational integration. This distinction is important because each domain addresses different questions, uses different data inputs, and permits different levels of inference, ranging from population-level surveillance and patient-level risk stratification to decision support and implementation-facing process improvement. AI has increasingly been proposed for infection surveillance, early risk stratification, antimicrobial decision support, and workflow-facing operational functions in acute-care settings (Shenoy and Branch-Elliman, 2023; Branch-Elliman et al., 2023; Talianu et al., 2026; Gastaldi et al., 2025). However, the evidence base remains methodologically uneven, clinically heterogeneous, and conceptually fragmented. Studies have examined diverse AI applications across surveillance support, risk prediction, infection detection, antimicrobial decision support, and workflow-facing functions, but these domains are not evidentially equivalent: they differ in their clinical purpose, data requirements, evaluation standards, and implications for practice (van Mourik et al., 2021; El Arab et al., 2025; Talianu et al., 2026). In particular, technical performance metrics should not be conflated with evidence of clinical effectiveness, prevention benefit, improved patient outcomes, or successful workflow integration. Accordingly, this scoping review did not seek to infer that reported AI performance translates into real-world effectiveness; rather, it aimed to map the literature, identify concentrations and gaps in the evidence, distinguish areas of relative translational maturity from persistent immaturity, and assess how far implementation-relevant evidence has accumulated.

### Aim

The aim of this scoping review was to map and synthesise the empirical literature on artificial-intelligence applications for infection surveillance, risk stratification, antimicrobial decision support, and workflow-relevant hospital functions, while explicitly distinguishing technical performance from clinical utility, implementation relevance, and patient benefit.

### Objectives

1. To examine how artificial intelligence has been studied across surveillance, risk prediction, infection detection, and management-support tasks relevant to infection prevention, infection control, and antimicrobial decision making in acute-care hospital settings.
2. To assess the extent to which included studies moved beyond technical performance to report implementation-relevant outcomes, including workflow fit, operational feasibility, real-time use, workload implications, staff-facing deployment, adoption-related signals, and clinically meaningful utility.

## Methods

### Study design

We undertook a scoping review to identify, map, and synthesise empirical studies evaluating artificial-intelligence applications relevant to infection surveillance, risk stratification, infection detection, antimicrobial decision support, and implementation-relevant workflow functions in acute-care hospital settings. A scoping-review design was considered appropriate because the evidence base was expected to be heterogeneous across target conditions, application domains, study designs, and evaluation endpoints; accordingly, the principal aim was evidence mapping and conceptual clarification rather than quantitative effect estimation. The review was conducted using established scoping-review methodological guidance and reported in accordance with the PRISMA extension for Scoping Reviews (PRISMA-ScR) (Munn et al., 2018; Tricco et al., 2018), with applicable elements of PRISMA 2020 used to support transparent reporting of study identification and selection (Page et al., 2021). Although the review was not prospectively registered, the review question, eligibility criteria, databases, search concepts, screening approach, extraction fields, and synthesis structure were specified before full-text screening. The absence of protocol registration reflected the scoping and exploratory nature of the review, but the conduct and reporting of the review were guided by PRISMA-ScR and relevant elements of PRISMA 2020 to maximise transparency and reproducibility. The absence of a registered protocol is acknowledged as a limitation.

### Search strategy and conceptual framing

We searched CINAHL, Cochrane Library, Embase, PubMed, Scopus, and Web of Science. Search concepts were developed through background reading and consultation of Medical Subject Headings used in MEDLINE and were combined with Boolean operators to maximise both sensitivity and conceptual breadth. The search strategy was structured around four domains: acute-care hospital setting, infection-related relevance, AI application type, and implementation or workflow relevance. Because the literature encompasses analytically distinct uses of AI, the conceptual framework was defined *a priori* according to application domain, target condition, stage of evaluation, and reported outcomes. Table 1 summarises the review objectives and conceptual framing used to organise the synthesis.

**Table 1:** Review objectives and conceptual framing.

Objective	Primary focus	Population and setting	AI application domains	Outcomes of interest
Objective 1	Surveillance, prediction, detection, and management-support applications relevant to infection prevention, infection control, and antimicrobial decision making in acute-care hospital settings	Hospitalised patients in acute-care hospital settings	Surveillance support; risk prediction; infection detection; management support; and antimicrobial decision support	Predictive performance; detection accuracy; classification performance; decision-support relevance; and infection-related clinical outcomes, where reported
Objective 2	Implementation-relevant and workflow-proximal evidence relating to the use of artificial intelligence in infection-control and hospital workflow contexts	Hospital-based healthcare professionals, infection-control activities, and workflow processes in hospital settings	Surveillance automation; prescribing support; real-time operational use; staff-facing implementation; behavioural monitoring; and related workflow-proximal applications	Operational feasibility; usability; decision-support relevance; manual workload reduction; workflow relevance; staff practice; real-time use; and implementation-related outcomes

For the purposes of synthesis, studies were grouped into two broad categories: first, infection surveillance, risk prediction, detection, and management-support applications; and second, implementation-relevant or workflow-facing applications, including surveillance automation, prescribing support, operational deployment, and staff-facing use.

The first objective review concerned surveillance, prediction, detection, and management-support applications relevant to infection prevention, infection control, and antimicrobial decision making in acute-care hospital settings. The second concerns implementation-relevant and workflow-proximal evidence, including surveillance processes, prescribing support, operational use, and staff-facing application in hospital settings. Table 2 presents the principal search concepts and exemplar terms used to operationalise the search strategy.

**Table 2:** Search concepts and exemplar terms used across the two review objectives.

Concept domain	Objective 1 exemplar terms	Objective 2 exemplar terms
Setting and population	“Hospitalised patients,” “inpatients,” “patients at risk of infection,” “hospital admission”	“Healthcare professionals,” “nurses,” “physicians,” “clinicians,” “infection-control teams,” “workflow,” “hospital setting”
AI methods	“Artificial intelligence,” “AI,” “machine learning,” “deep learning,” “neural networks”	“Artificial intelligence,” “AI,” “machine learning,” “clinical decision support,” “surveillance automation,” “real-time systems”
Infection focus	“Healthcare-associated infection,” “hospital-acquired infection,” “surgical site infection,” “ventilator-associated pneumonia,” “urinary tract infection,” “bacteraemia,” “antimicrobial resistance,” “infection management”	“Infection control,” “infection surveillance,” “antimicrobial prescribing,” “staff monitoring,” “infection-control practice,” “decision support”
Outcomes and implementation	“Prediction,” “detection,” “risk stratification,” “classification,” “surveillance,” “management support,” “patient safety”	“Implementation,” “workflow-proximal,” “adoption,” “usability,” “manual workload,” “decision-making,” “real-time use,” “operational feasibility”

The complete database-specific search strategies, including Boolean operators, field tags, truncation, date limits, and database-specific adaptations, are provided in Supplementary Appendix 1.

### Eligibility criteria and study selection

Eligibility criteria were applied consistently across both search strategies and are summarised in Table 3. Eligible studies were English-language publications reporting original empirical quantitative, qualitative, or mixed-methods data relevant to at least one review objective and published between Jan 1, 2016, and Jan 31, 2026. Searches were last run on Feb 1, 2026, with identical date limits applied across all databases.

**Table 3:** Eligibility criteria.

Category	Inclusion criteria	Exclusion criteria
Study design	Primary empirical studies reporting original quantitative, qualitative, or mixed-methods data relevant to one or both review objectives	Systematic reviews, meta-analyses, narrative or other literature reviews, editorials, opinion pieces, conference abstracts without sufficient data, and theoretical or conceptual papers without original empirical findings
Population and setting	Hospitalised patients and/or hospital-based healthcare professionals in acute-care hospital settings	Community-only settings, outpatient-only settings, and non-healthcare settings
AI application	Artificial-intelligence applications relevant to infection surveillance, prediction, detection, management support, antimicrobial decision making, infection-control processes, or implementation-relevant use in hospital settings	AI applications unrelated to infection prevention, infection control, infection management, antimicrobial decision support, or hospital-based operational use
Outcomes	Infection-related predictive, diagnostic, management-support, implementation-relevant, or workflow-proximal outcomes relevant to the review objectives	Studies not reporting outcomes relevant to infection control, clinical decision making, surveillance, patient safety, antimicrobial decision support, or implementation-related practice
Language	English-language publications	Non-English-language publications
Publication date	Studies published between Jan 1, 2016, and Jan 31, 2026	Studies published outside the predefined period

Duplicate records were removed after database searching. Titles and abstracts were screened against predefined eligibility criteria by two reviewers, with uncertain records retained for full-text assessment. Full texts were then assessed for eligibility using the same criteria. Disagreements or uncertainties at either stage were resolved through discussion, with consultation of a senior reviewer when consensus was not reached. Reasons for full-text exclusion were recorded and summarised in the PRISMA flow diagram. Study selection was therefore conducted using predefined criteria and consensus procedures to reduce the risk of arbitrary inclusion or exclusion (Tricco et al., 2018; Page et al., 2021).

### Data extraction and synthesis

Data were extracted using a standardised extraction framework developed for this review. Extracted items included author, year, country, setting, population, sample size, study design, data source, AI method, target infection-related condition or task, outcome definition, validation approach, performance metrics, comparator where available, implementation-relevant outcomes, and principal findings. Extracted data were checked for completeness and consistency by a second reviewer, with discrepancies resolved by discussion. Where reported, we distinguished internal validation, external validation, prospective evaluation, real-time deployment, and implementation-facing evaluation (Munn et al., 2018; Peters et al., 2021; Peters et al., 2020). Because this was a scoping review of a heterogeneous evidence base rather than a review designed to generate pooled estimates of effect, we did not apply a single formal risk-of-bias instrument across all included studies (Munn et al., 2018; Tricco et al., 2018; Peters et al., 2021; Peters et al., 2020). The included literature varied substantially in clinical focus, study design, data source, AI method, validation approach, target outcome, and stage of evaluation, making a single conventional appraisal tool unlikely to capture the methodological issues most relevant to this field. Instead, methodological credibility and translational maturity were assessed narratively during data extraction and synthesis.

This structured narrative appraisal considered whether studies used retrospective or prospective designs; whether datasets were single-centre or multicentre; whether outcome definitions and reference standards were clearly described; whether models were internally, externally, temporally, or prospectively validated; whether discrimination, calibration, clinical utility, and comparator performance were reported; and whether studies assessed workflow consequences, usability, adoption, clinician trust, implementation feasibility, or patient-level outcomes. These domains were not used to exclude studies, in keeping with the scoping-review aim of mapping the available literature, but they informed the interpretation of the strength, maturity, and applicability of the evidence. Studies were therefore interpreted cautiously, with particular attention to the distinction between technical model performance, implementation readiness, and demonstrated clinical benefit.

## Results

### Findings

**Figure 1:**
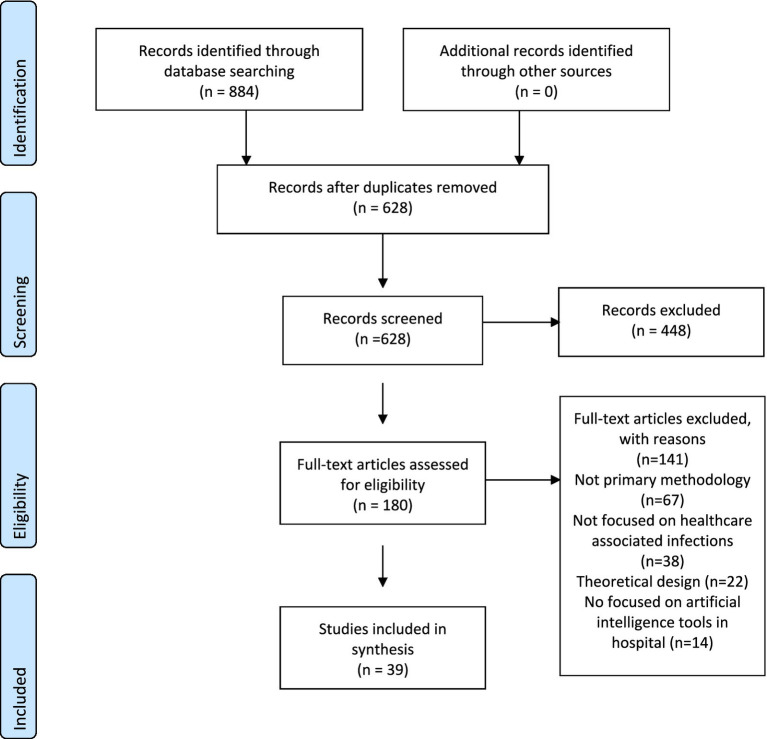
PRISMA flow diagram.

The database searches yielded 884 records. After removal of duplicates, 628 unique records remained for title and abstract screening, of which 180 articles underwent full-text assessment and 39 studies were included in the final synthesis (Figure 1). The principal reasons for exclusion were lack of an original empirical study design, insufficient focus on healthcare-associated infections, or limited relevance to AI-related outcomes. The included literature contained no randomised trials or other robust comparative evaluations of AI under routine clinical conditions. Rather, the evidence base was composed predominantly of retrospective cohort studies for model development and validation, supplemented by retrospective or cross-sectional comparisons with clinical datasets or conventional clinical markers.

The search relating to the first review objective identified 37 studies (Wu et al., 2023; Sophonsri et al., 2023; Rafaqat et al., 2023; Mamlook Al et al., 2023; Kiser et al., 2023; Jakobsen et al., 2024a; Chen et al., 2023; Cai et al., 2023; Bonde et al., 2023; Zhu et al., 2022; Scala et al., 2022; Rennert-May et al., 2022; Ohno et al., 2022; Liu et al., 2022; Li et al., 2022; Caǧlayan et al., 2022; Wang et al., 2021; Walker et al., 2021; Petrosyan et al., 2021; Møller et al., 2021; Lind et al., 2021; Giang et al., 2021; dos Santos et al., 2021; Barchitta et al., 2021; Hopkins et al., 2020; Zachariah et al., 2020; Mancini et al., 2020; Chen et al., 2020; Liao et al., 2019; Sohn et al., 2017; Jakobsen et al., 2024b; Cho et al., 2024; Bolton et al., 2024; Tsai et al., 2023; Huang et al., 2023; Flores-Balado et al., 2023; Kanjilal et al., 2020), whereas nine studies contributed to the second objective (Jakobsen et al., 2024b; Cho et al., 2024; Bolton et al., 2024; Tsai et al., 2023; Huang et al., 2023; Flores-Balado et al., 2023; Kanjilal et al., 2020; Yelin et al., 2019; Oonsivilai et al., 2018). Because some studies were relevant to both objectives, 39 unique studies were included overall. In view of the considerable methodological heterogeneity of the included literature, findings were synthesised narratively and interpreted cautiously, particularly where studies were characterised by retrospective designs, small sample sizes, limited validation, or sparse evaluation of implementation outcomes. The structured narrative appraisal showed that most studies remained at an early translational stage, with evidence concentrated around retrospective model development or validation, limited external or prospective evaluation, inconsistent reporting of calibration and clinical utility, and sparse assessment of workflow integration or patient-level benefit. The remainder of the Results section is presented thematically in accordance with the two predefined review objectives.

### Artificial intelligence for surveillance, prediction, detection, and management-support applications in infection-related hospital contexts

A total of 37 studies contributed to the evidence base for the first review objective and are summarised in Table 4. Overall, the included literature focused predominantly on AI applications for prediction, classification, detection, surveillance support, and risk stratification, rather than on direct prevention effects or demonstrated improvements in patient outcomes. Accordingly, this body of evidence is more appropriately interpreted as evidence of technical or diagnostic performance than as definitive evidence of clinical effectiveness. Across studies, the most frequently reported performance metrics were area under the receiver operating characteristic curve, sensitivity, specificity, and predictive values. The evidence clustered around surgical-site infections, urinary-tract-infection-related outcomes, ventilator-associated pneumonia, and a smaller but heterogeneous group of broader healthcare-associated-infection-related outcomes, including sepsis, bacteraemia, resistant organisms, recurrence, and infection-related mortality.

**Table 4:** Summary of included studies evaluating artificial-intelligence applications for infection surveillance, risk stratification, detection, antimicrobial decision support, and related hospital applications.

Author and date	Setting	Sample	AI tool and methodology	Target condition or application domain	Key findings
Sohn et al. (2017)	General surgery	Patients undergoing colorectal cancer surgery	Bayesian network identifying risk factors from a quality-improvement programme and keywords from clinical notes	SSI	SSI detection yielded an ROC AUC of 0.827, increasing to 0.892 when surgeon-defined clinically meaningful SSIs were used
Liao et al. (2019)	Intensive care unit	Patients receiving ventilator treatment	Artificial neural network and support vector machine using sensor-array signals derived from patient records (*n* = 140)	VAP due to *Pseudomonas aeruginosa*	Artificial neural network and support vector machine models achieved AUCs of 0.984 and 0.941, with high sensitivity and positive predictive values
Chen et al. (2020)	ICU	Patients receiving ventilator treatment, with controls	Machine learning applied to electronic-noise sensor-array signals	VAP	Across eight machine-learning algorithms, mean accuracy was 0.81, sensitivity was 0.79, specificity was 0.83, PPV was 0.85, NPV was 0.77, and AUC was 0.85
Mancini et al. (2020)	Hospital	All admitted patients	Machine-learning model trained on 1,486 patients	Multidrug-resistant UTI	Best-performing algorithm achieved an AUC of 0.739 and diagnostic accuracy of 0.717
Zachariah et al. (2020)	Hospital	All patients admitted to hospital	Neural-network and decision-tree models using admission EHR data (*n* = 897,344)	UTI risk prediction	The decision-tree model showed higher sensitivity than the neural-network model (78.2% vs. 57.3%) but lower specificity (64.2% vs. 81.4%); NPVs exceeded 99% for both models
Hopkins et al. (2020)	Orthopaedic and neurological surgery	Patients undergoing posterior spinal fusion surgery	Neural-network model trained on 4,046 patients	SSI	Predicted SSIs with an AUC of 0.775, PPV of 92.56%, and NPV of 98.45%; also identified risk and protective factors
Kanjilal et al. (2020)	Hospital	Patients with UTIs	Machine-learning model generating antibiotic recommendations for UTI using 10,053 patients	Antimicrobial decision support for UTI	The model generated a recommendation in 99% of cases
Barchitta et al. (2021)	ICU	All admitted patients	Machine-learning model for ICU-associated infection risk using 20,060 patients	HAI risk prediction	AUC was 0.90 when combined with existing risk-stratification tools
Dos Santos et al. (2021)	Hospital	All admitted patients	Neural-network model for HAI monitoring using 18 months of data (5,105 patients)	HAI surveillance	ROC AUC was 0.903, sensitivity was 88.57%, and specificity was 90.27%; the model correctly classified 67 of 73 patients with HAIs
Giang et al. (2021)	ICU	Ventilated adults	Machine-learning model trained on 6,126 adults	VAP	The best-performing model produced an AUC of 0.854; predictive features included ventilation duration, antibiotic use, sputum testing, and Glasgow Coma Scale variables
Lind et al. (2021)	Hospital	Recipients of allogeneic haematopoietic cell transplant	Two automated machine-learning systems using EHR data from 1943 transplant recipients	Sepsis / bacteraemia	Systems showed sensitivities of 80.0 and 65.7%, and specificities of 72.8 and 66.9%, respectively
Møller et al. (2021)	Hospital	All admitted patients	Decision-tree models using demographics, laboratory, antibiotic, and clinical data from 301,932 patients	HAI-related UTI risk prediction	Both models achieved ROC values of 0.74 and 0.81 and supported personalised UTI-prevention strategies
Petrosyan et al. (2021)	Hospital	Patients undergoing surgery	Machine-learning model for SSI prediction based on 14,351 patients	SSI	Model achieved an AUC of 0.91
Walker et al. (2021)	Paediatric ward	Children with CLABSI	Machine-learning model predicting outcomes of CVC salvage using EHR data from 969 children	HAI recurrence	Demonstrated superior prediction of recurrence compared with CVC-removal models, with AUCs of 0.83 and 0.77 versus 0.66 and 0.76
Wang et al. (2021)	General surgery	Patients undergoing minimally invasive surgery	Machine-learning models using clinical and laboratory data from 705 patients	SSI	Bayesian algorithm achieved an AUC of 0.78
Caǧlayan et al. (2022)	ICU	All ICU admissions	Data-driven framework for predicting MRSA colonisation using 3,958 ICU patients	MRSA colonisation/HAI risk	Best-performing models showed sensitivity of 73%–80% and specificity of 59%–83%
Li et al. (2022)	ICU	Patients with candidiasis and bloodstream bacterial infection	Random-forest and other machine-learning models using 246 hospitalised patients	Infection-related mortality	The random-forest model achieved an AUC of 0.919 and identified important mortality risk factors
Liu et al. (2022)	Hospital	Patients undergoing lumbar spinal surgery	Machine-learning model using 288 patients	SSI	Best-performing model achieved an AUC of 0.926
Ohno et al. (2022)	General surgery	Patients undergoing surgery for colon cancer	Prediction model using immunological and nutritional markers trained on 730 patients	SSI	Model achieved an AUC of 0.73 and performed comparably with alternative risk models
Rennert-May et al. (2022)	Hospital	Patients receiving cardiac devices	Machine-learning model using 3,536 procedures	SSI	Achieved an AUC of 0.968
Scala et al. (2022)	Hospital	Surgical patients excluding those with diabetes or corticosteroid therapy	Logistic-regression model using EHR data from 4,031 patients	SSI	Best-performing model showed sensitivity of 95.9%, specificity of 94.9%, and high overall accuracy
Zhu et al. (2022)	Hospital	Immobile patients after stroke	Predictive models using 3,892 patients	HAI-related UTI risk	Best ensemble-learning model achieved an ROC AUC of 0.822 and sensitivity of up to 81.1%
Bonde et al. (2023)	Surgery	Patients undergoing surgery	Natural-language-processing model using EHR chart notes from 389,865 surgical cases	SSI	Outperformed administrative data, with sensitivity of 60.4–85.4%, specificity of 98.7–99.6%, and high predictive values
Cai et al. (2023)	Hospital	Women with recurrent UTI	Artificial-neural-network model trained on 725 patients and validated on 318 patients	Prediction of antibiotic efficacy in recurrent UTI	Sensitivity was 87.8% and specificity was 97.3% for predicting efficacy of empirical antibiotic therapy
Chen et al. (2023)	Gastrointestinal surgery	Patients undergoing colorectal surgery	Deep-neural-network model using 275,152 patient records	SSI	ROC AUC was 0.769, specificity was 50.0%, and sensitivity was 82.0%
Flores-Balado et al. (2023)	Orthopaedic surgery	Patients undergoing hip replacement surgery	Natural-language-processing and extreme-gradient-boosting model using EHR data from 6,741 patients	SSI	Sensitivity was 99.18% and specificity was 91.0%
Huang et al. (2023)	Hospital	Hospital staff	Camera and speaker systems with integrated AI monitors for PPE and handwashing training	Staff-facing infection-control monitoring	System accuracy increased from 52.15 to 98.14%, with infection rate decreasing from 1.31% to 0.38%
Jakobsen et al. (2024a)	Hospital	All admitted patients	Machine-learning model for UTI prediction from admission EHR records using 138,650 patients	HAI-related UTI risk prediction	Best-performing deep-neural-network model achieved an AUC of 0.758 and identified high-risk patients within 24 h of admission
Kiser et al. (2023)	General surgery	All surgical patients	Long short-term memory model based on EHR data from 9,185 patients	SSI	Maximum ROC AUC was 0.905
Mamlook Al et al. (2023)	General surgery	Patients undergoing surgery	Deep-neural-network model using 2,882,526 patient records	SSI	AUC was 0.8518, accuracy was 0.8518, and sensitivity was 0.8527
Rafaqat et al. (2023)	Surgery	Patients undergoing any surgery	Machine-learning model predicting SSI type and timing using 113 patients	SSI	Best model achieved an AUC of 0.84 for SSI type and 0.74 for timing of SSI prediction
Sophonsri et al. (2023)	Hospital	Patients with pneumonia	Machine-learning models identifying mortality risk factors using 457 patients	Infection-related mortality in pneumonia	AUC values ranged from 0.83 to 0.90 for different pneumonia-related mortality models
Tsai et al. (2023)	Emergency department	Febrile adults	Random-forest and logistic-regression models trained on 3,669 patients	Bacteraemia prediction	Best random-forest model achieved an AUC of 0.761, outperforming the clinical scoring system (0.560)
Wu et al. (2023)	Hospital	Patients undergoing hip or knee arthroplasty	Machine-learning model trained on 22,059 patients	SSI	Best model achieved an ROC AUC of 0.906 and sensitivity of 83.9%
Bolton et al. (2024)	ICU	All ICU admissions	Machine-learning model predicting when patients can switch from intravenous to oral antibiotics based on 10,362 ICU stays	Antimicrobial management support	ROC AUC was 0.80 and the model supported IV-to-oral switch decision making
Cho et al. (2024)	Hospital	Patients undergoing colorectal surgery	Deep-neural-network models using 1,652 surgical cases	SSI surveillance	Deep-neural-network model achieved an AUC of 0.963 and PPV of 21.1%; PPV increased to 28.9% when combined with a rule-based algorithm
Jakobsen et al. (2024a)	Hospital	All admitted patients	Bayesian-network model using 138,250 admissions and 50 features	HAI-related UTI risk prediction	Five-feature model achieved an AUC of 0.746 and supported early risk stratification within 24 h of admission

AUC, area under the curve; CLABSI, central line-associated bloodstream infection; CVC, central venous catheter; EHR, electronic health record; HAI, healthcare-associated infection; ICU, intensive care unit; IV, intravenous; MRSA, meticillin-resistant *Staphylococcus aureus*; NPV, negative predictive value; PPE, personal protective equipment; PPV, positive predictive value; ROC, receiver operating characteristic; SSI, surgical-site infection; UTI, urinary-tract infection; VAP, ventilator-associated pneumonia.

### Surgical-site infections

Surgical-site infections were the most frequently studied infection group, with 16 studies examining this area across diverse surgical settings, including colorectal, spinal, orthopaedic, lumbar, cardiac-device, arthroplasty, and mixed surgical populations (Wu et al., 2023; Sophonsri et al., 2023; Rafaqat et al., 2023; Mamlook Al et al., 2023; Kiser et al., 2023; Chen et al., 2023; Bonde et al., 2023; Rennert-May et al., 2022; Ohno et al., 2022; Liu et al., 2022; Wang et al., 2021; Petrosyan et al., 2021; Hopkins et al., 2020; Sohn et al., 2017; Cho et al., 2024; Flores-Balado et al., 2023). Collectively, these studies suggested that artificial-intelligence models could identify relevant risk factors, stratify patients according to postoperative infection risk, or support detection of surgical-site infections, with generally moderate-to-high discriminatory performance reported across datasets. Sohn et al. (2017) reported that a Bayesian-network model identified clinically important colorectal surgical-site infections with an AUC of 0.827, increasing to 0.892 when surgeon-defined clinically meaningful infections were used. Other studies likewise reported promising discrimination for postoperative surgical-site-infection prediction, including AUC values of 0.91 in Petrosyan et al. (2021), 0.926 in Liu et al. (2022), 0.968 in Rennert-May et al. (2022), and 0.906 in Wu et al. (2023). Natural-language-processing and deep-learning approaches also showed encouraging performance for surveillance and detection, as illustrated by the studies by Bonde et al. (2023); Cho et al. (2024); Flores-Balado et al. (2023). The surgical-site-infection literature suggests substantial promise for artificial intelligence in risk prediction and surveillance support within surgical populations, although the evidence remains dominated by retrospective and internally validated studies.

### Urinary-tract-infection-related outcomes

Several included studies addressed urinary-tract-infection-related outcomes, although they varied substantially in clinical context, target outcome, and degree of relevance to hospital-acquired-infection prevention (Jakobsen et al., 2024a; Cai et al., 2023; Zhu et al., 2022; Møller et al., 2021; Zachariah et al., 2020; Mancini et al., 2020; Jakobsen et al., 2024b; Kanjilal et al., 2020). Some studies focused on the early identification of patients at risk of hospital-acquired urinary tract infection using admission data. Zachariah et al. (2020) showed that admission-based models could identify patients at risk of urinary tract infection at the point of hospital admission, with a decision-tree model demonstrating higher sensitivity but lower specificity than the neural-network model. Møller et al. (2021) similarly reported ROC values of 0.74 and 0.81 for models based on admission and historical data, with the aim of informing more individualised preventive strategies. The studies by Jakobsen et al. (2024a, 2024b) further suggested that machine-learning and Bayesian-network approaches derived from admission data could identify patients at elevated risk of hospital-acquired urinary tract infection within the first 24 h of admission. By contrast, other studies addressed multidrug-resistant urinary tract infection, post-stroke urinary tract infection risk, and prediction of antibiotic efficacy in recurrent urinary tract infection, rather than hospital-acquired urinary tract infection risk itself (Cai et al., 2023; Zhu et al., 2022; Mancini et al., 2020). These differences warrant cautious interpretation and should not be treated as constituting a single, homogeneous body of evidence on hospital-acquired urinary-tract-infection prevention.

### Ventilator-associated pneumonia

Three studies focused on ventilator-associated pneumonia in intensive-care populations (Chen et al., 2023; Giang et al., 2021; Liao et al., 2019). Liao et al. (2019) applied artificial neural networks and support vector machines to electronic-noise sensor-array data and reported AUC values of 0.984 and 0.941, respectively. Chen et al. (2023) reported mean accuracy of 0.81, sensitivity of 0.79, specificity of 0.83, and an AUC of 0.85 across eight machine-learning algorithms. Giang et al. (2021) found that the best-performing model achieved an AUC of 0.854, with influential predictors including duration of ventilation, antibiotic use, sputum-test frequency, and Glasgow Coma Scale variables. This literature suggests that AI approaches may support earlier recognition of ventilator-associated pneumonia in high-risk mechanically ventilated patients, although the available evidence remains confined to retrospective or cross-sectional evaluation rather than prospective implementation.

### Other healthcare-associated-infection-related outcomes

Several studies addressed a broader and more heterogeneous range of infection-related outcomes, including general healthcare-associated infections at hospital or intensive-care-unit admission, recurrence of infection, sepsis and bacteraemia risk, colonisation with resistant organisms, infection-related mortality, pneumonia, and staff-facing infection-prevention monitoring. Barchitta et al. (2021) reported an AUC of 0.90 for prediction of ICU-associated infection risk when combined with existing risk-stratification tools. dos Santos et al. (2021) described an automated neural-network-based surveillance model for healthcare-associated infections with an AUC of 0.903, sensitivity of 88.57%, and specificity of 90.27%. Lind et al. (2021) found that automated machine-learning systems showed moderate sensitivity and specificity for predicting sepsis and bacteraemia among recipients of stem-cell transplant. Tsai et al. (2023) reported that a real-time bacteraemia prediction model outperformed a clinical scoring system, with an AUC of 0.761 compared with 0.560. Huang et al. (2023), although more directly relevant to workflow and behavioural monitoring, also reported reduced infection rates alongside improved compliance with personal protective equipment use and handwashing guidance. Overall, these studies suggest that AI methods may have broad technical applicability across a range of infection-related tasks, while also highlighting substantial heterogeneity in target conditions, datasets, and intended use cases.

### Implementation-relevant and workflow-proximal evidence relating to artificial intelligence in infection-control contexts

A total of nine studies contributed to the second review objective and are summarised in Table 5. Compared with the literature on technical performance, the evidence relating to implementation and workflow was both more limited and more heterogeneous. Most studies addressed decision-support relevance, prescribing support, surveillance workload reduction, operational feasibility, or staff-facing implementation, rather than more comprehensive implementation outcomes such as clinician adoption, usability, trust, workflow redesign, sustained real-world effectiveness, or patient benefit. This body of evidence is therefore better interpreted as implementation-relevant or workflow-proximal than as definitive evidence of successful workflow integration (Jakobsen et al., 2024b; Cho et al., 2024; Bolton et al., 2024; Tsai et al., 2023; Huang et al., 2023; Flores-Balado et al., 2023; Kanjilal et al., 2020; Yelin et al., 2019; Oonsivilai et al., 2018).

**Table 5:** Summary of included studies evaluating implementation-relevant and workflow-proximal applications of artificial intelligence in infection-control and hospital workflow contexts.

Author and date	Setting	Sample	AI tool and methodology	Implementation or workflow domain	Key workflow-related finding
Kanjilal et al. (2020)	Hospital	Patients with UTIs	Machine-learning model generating antibiotic recommendations for UTI using 10,053 patients	Antimicrobial decision support	The model generated a recommendation in 99% of cases, suggesting feasibility for routine prescribing support
Flores-Balado et al. (2023)	Orthopaedic surgery	Patients undergoing hip replacement surgery	Natural-language-processing and extreme-gradient-boosting model using EHR data from 6,741 patients	SSI surveillance and manual workload reduction	Used as a screening-support tool for SSI surveillance and reduced the volume of records requiring manual review across four hospitals
Huang et al. (2023)	Hospital	Hospital staff	Camera and speaker systems with integrated AI monitoring for PPE and handwashing training	Staff-facing implementation and behavioural monitoring	Reported improvements in hand hygiene and PPE use, with a concurrent reduction in infection rates; however, causal attribution to the AI system cannot be established from the study design
Tsai et al. (2023)	Emergency department	Febrile adults	Real-time random-forest and logistic-regression bacteraemia prediction system using 3,669 patients	Real-time decision support	Real-time prediction performed more favourably than clinical risk scoring and could reduce unnecessary empirical antibiotic prescribing
Bolton et al. (2024)	ICU	All ICU admissions	Machine-learning model for IV-to-oral antibiotic switch decisions based on 10,362 ICU stays	Antimicrobial workflow support	Supported decision making for intravenous-to-oral antibiotic switching and could reduce unnecessary intravenous antibiotic use
Cho et al. (2024)	Hospital	Patients undergoing colorectal surgery	Deep-neural-network model combined with a rule-based algorithm using 1,652 surgical cases	SSI surveillance workload reduction	Reduced the number of new cases requiring review compared with conventional surveillance practice
Jakobsen et al. (2024b)	Hospital	All admitted patients	Bayesian-network risk-stratification model within 24 h of admission	Early operational risk stratification	Supported early operational risk stratification by identifying patients at elevated UTI risk within the first 24 h of admission, although implementation features were not directly evaluated
Oonsivilai et al. (2018)	Paediatric hospital	Patients with infection requiring empiric antibiotic therapy	Models to guide targeted and locally tailored antibiotic prescribing	Antibiotic selection and prescribing support	Random-forest model achieved an AUC of 0.80 for predicting antibiotic susceptibility and could support locally tailored prescribing decisions
Yelin et al. (2019)	Hospital	Patients with UTIs	Model using clinical history to predict antibiotic resistance	Antibiotic resistance prediction and prescribing support	AUC was 0.70 for amoxicillin and 0.80 for ciprofloxacin; mismatch between prescribed antibiotics and resistance profiles was reduced by 42% compared with physician prescribing

AUC, area under the curve; EHR, electronic health record; ICU, intensive care unit; IV, intravenous; PPE, personal protective equipment; SSI, surgical-site infection; UTI, urinary-tract infection.

### Prescribing support and treatment decision-making

Several studies suggested that artificial-intelligence models may support antibiotic selection or prescribing decisions. Oonsivilai et al. (2018) reported that a random-forest model could guide targeted and locally tailored antibiotic prescribing in a paediatric setting, with an AUC of 0.80 for predicting susceptibility to selected antibiotics. Yelin et al. (2019) found that a model based on clinical history could predict antibiotic resistance in urinary-tract infections and reduce mismatch between prescribed antibiotics and resistance profiles compared with physician prescribing. Kanjilal et al. (2020) reported a lower risk of inappropriate antibiotic prescribing than physicians in uncomplicated urinary-tract infection, suggesting potential value as a prescribing-support tool. Bolton et al. (2024) similarly reported that a machine-learning model could support decisions regarding switching from intravenous to oral antibiotics in intensive care. These studies suggest potential operational value for antimicrobial decision support, but they do not establish broader workflow transformation or patient-level benefit.

### Surveillance workload reduction and operational relevance

Other studies suggested that artificial intelligence may reduce time spent on selected infection-control-related tasks or improve operational efficiency. Flores-Balado et al. (2023) reported that an artificial-intelligence-based screening tool for surgical-site infections reduced the volume of records requiring manual review across four hospitals. Tsai et al. (2023) found that a real-time bacteraemia prediction model performed more favourably than a clinical scoring system and could reduce unnecessary empirical antibiotic prescribing. Cho et al. (2024) reported that a deep-neural-network model, when combined with a rule-based algorithm, reduced the number of new cases requiring review in colorectal surgical-site-infection surveillance. Jakobsen et al. (2024b) suggested that Bayesian-network-based early risk stratification could support timely decision making for urinary-tract-infection prevention within the first 24 h of admission, although implementation features were not directly evaluated. Kanjilal et al. (2020) also showed that algorithm-generated antibiotic recommendations could be produced in 99% of uncomplicated urinary-tract-infection cases, which suggests feasibility for decision support in routine prescribing contexts. Collectively, these studies indicate operational relevance but remain insufficient to demonstrate comprehensive implementation success across routine infection-control workflows.

### Staff-facing implementation and behavioural monitoring

One study differed substantially from the rest of the workflow literature by focusing on staff-facing behavioural intervention rather than on prediction or prescribing support. Huang et al. (2023) evaluated an AI-based training and monitoring system using camera and speaker systems to guide personal protective equipment use and handwashing among hospital staff. The study reported improved system accuracy over time together with a concurrent reduction in infection rates (Huang et al., 2023), although distinctive and potentially important, this study was unique within the included literature, and comparable evaluations of staff-monitoring systems were not identified elsewhere in the review.

## Discussion

This scoping review addressed two related but analytically distinct questions: first, how AI has been studied across infection-related surveillance, risk prediction, detection, and management-support functions in acute-care hospital settings; and second, the extent to which these applications have been evaluated beyond technical accuracy in implementation-relevant, workflow-facing, or clinically meaningful terms. Across 39 included studies, the literature was substantially more developed for retrospective technical evaluation than for prospective translational evaluation. The central message of this review is therefore not simply that AI shows promise, but that the field remains fundamentally unbalanced: technical performance has been studied much more extensively than clinical utility, implementation, and patient benefit.

The first objective incorporated 37 studies spanning surgical-site infections, urinary-tract-infection-related outcomes, ventilator-associated pneumonia, bacteraemia, sepsis, resistant organisms, recurrence, and infection-related mortality. Collectively, these studies indicate that AI has emerging technical potential for risk stratification, prediction, surveillance support, and selected management-support tasks. Nevertheless, such promises should be interpreted cautiously. The evidence base remains dominated by retrospective model-development and validation studies, with limited prospective implementation, sparse reporting of patient-level outcomes, and restricted evaluation of clinical utility beyond statistical discrimination.

The second objective concerned implementation-relevant and workflow-proximal applications of AI in infection-control contexts. Far fewer studies examined this dimension directly, and none explored barriers or facilitators to the use of AI in practice from the perspective of doctors, nurses, or other healthcare professionals. The available evidence points to possible operational relevance in areas such as prescribing support, reduction of surveillance workload, and selected decision-support functions, but it remains insufficient to justify strong conclusions regarding full workflow integration, successful implementation, or wider improvement in infection-control practice.

The value of surveillance strategies for healthcare-associated infections, including those relevant to prevention, diagnosis, and management, has long been recognised, and the use of emerging technologies to strengthen surveillance has increasingly been advocated (Liao et al., 2019). Only recently, however, have AI technologies matured to a degree that suggests a potentially meaningful shift in how surveillance is conducted and how decisions are made in relation to healthcare-associated infections (Advani et al., 2024). Earlier systematic reviews suggested that electronic and automated tools could support the surveillance and monitoring of healthcare-associated infections (Roel Streefkerk et al., 2020; Russo et al., 2018). However, this literature is now partly outdated given rapid developments in machine learning, artificial intelligence, electronic health-record infrastructure, and the growing diversity of available models (El Arab et al., 2025; Scardoni et al., 2020; Cozzolino et al., 2025).

A systematic review by Radaelli et al. (2024) suggested that AI, and machine learning in particular, may support healthcare staff through earlier identification of healthcare-associated infections and through prevention-oriented evaluation of personalised risk factors. Reported benefits included possible reductions in cost, time, and workload, together with potential gains in accuracy relative to conventional practice (Radaelli et al., 2024). However, that review incorporated multiple healthcare settings rather than hospital settings alone, which limits direct comparability with the present synthesis (Radaelli et al., 2024). More importantly, much of the underlying literature still lacks clear evidence of implementation in routine clinical practice, demonstrable patient benefit, or sustained workflow integration. Although reported model performance is often promising, few studies have evaluated real-world effects on patient outcomes, workload, costs, or infection-prevention processes (El Arab et al., 2025; Cozzolino et al., 2025; Baddal et al., 2024).

Risk stratification can be informed by numerous clinical variables, including demographic characteristics, comorbidities, invasive devices, antimicrobial exposure, admission type, length of stay, and markers of illness severity (Liu et al., 2023; Carestia et al., 2023; Rodríguez-Acelas et al., 2017). The findings of the present review emphasise the potential relevance of AI to infection-related risk stratification, surveillance support, and selected management-support tasks in hospital settings. Healthcare-associated infections remain an important source of morbidity and mortality, and several infection outcomes are difficult to identify or anticipate early in the course of hospital care, particularly when signals are distributed across clinical, microbiological, prescribing, device-use, and workflow data (Raoofi et al., 2023; Shenoy and Branch-Elliman, 2023; Branch-Elliman et al., 2023; El Arab et al., 2025).

AI techniques may support multidimensional analysis of routinely collected clinical data and enable more efficient identification of patients at elevated infection risk (Møller et al., 2021; Zachariah et al., 2020; Kanjilal et al., 2020). Related approaches have also been used to identify clinically relevant antimicrobial-resistance patterns from microbiological, genomic, prescribing, or electronic health-record data (Jakobsen et al., 2024a, 2024b; Bolton et al., 2024). However, most studies assessed technical or predictive performance rather than effects on infection incidence, patient outcomes, staff workload, antimicrobial use, or care processes under routine clinical conditions. These findings therefore support the operational promise of AI-enabled surveillance and decision support, but do not yet establish prevention effectiveness, clinical benefit, or superiority over existing care pathways.

The potential role of AI in guiding decision making in infection-control contexts is also important because effective antimicrobial therapy is central to reducing resistance, improving treatment outcomes, and minimising inappropriate treatment (Radaelli et al., 2024). Within the included literature, empirical antibiotic prescribing was vulnerable, in selected cases, to mismatch with subsequent resistance profiles or to inappropriate antibiotic selection. AI-based decision-support tools showed modest improvements in prescribing support, including reduced mismatch, improved identification of inappropriate prescriptions, or more guideline-concordant antibiotic selection (Cai et al., 2023; Kanjilal et al., 2020; Yelin et al., 2019; Oonsivilai et al., 2018). However, these benefits were generally modest, strongly context-dependent, and rarely evaluated at scale with patient outcomes as the primary endpoint. Further work is therefore required to validate such decision-support tools and to clarify whether they should replace, augment, or simply complement existing antimicrobial decision frameworks (Radaelli et al., 2024). The wider literature further indicates that integration of AI into infection-control workflows should be evaluated not only in terms of technical performance, but also in relation to feasibility, usability, trust, and the perspectives of healthcare staff (Tun et al., 2025; Bolton et al., 2025; Barbati et al., 2024; Aung et al., 2021). Implementation of AI-enabled infection-surveillance and decision-support tools is likely to depend on several interacting barriers and facilitators. Barriers include incomplete or delayed data capture, poor interoperability between electronic-health-record, microbiology, prescribing, and surveillance systems, unclear risk thresholds, limited explainability, alert fatigue, uncertain accountability, and insufficient evidence that model outputs lead to actionable infection-prevention or antimicrobial-stewardship decisions (van Mourik et al., 2021; Branch-Elliman et al., 2023; Gastaldi et al., 2025; Cozzolino et al., 2025; Tun et al., 2025; Aung et al., 2021; Vasey et al., 2022). Facilitators include local validation before deployment, prospective silent-mode testing, integration into existing infection-control workflows, transparent thresholds linked to clear action protocols, clinician involvement in design and evaluation, governance for monitoring and model updating, and mechanisms for withdrawing or modifying tools if performance deteriorates (van Mourik et al., 2021; Branch-Elliman et al., 2023; Gastaldi et al., 2025; Cozzolino et al., 2025; Tun et al., 2025; Barbati et al., 2024). These considerations reinforce the need to evaluate AI systems as sociotechnical interventions rather than as stand-alone predictive models (Gastaldi et al., 2025; Tun et al., 2025; Aung et al., 2021).

Research addressing the organisational and behavioural dimensions of implementation remains relatively limited, reflecting the novelty of deploying AI systems in these contexts (Radaelli et al., 2024; Gastaldi et al., 2025; Cozzolino et al., 2025). There is therefore a need for stronger prospective, head-to-head, and externally validated studies comparing AI-based approaches with conventional tools under real clinical conditions (Radaelli et al., 2024; Cozzolino et al., 2025; Barbati et al., 2024). Substantial heterogeneity also persists in the development and application of AI models for healthcare-associated-infection prevention and management, reflecting differences in underlying datasets, variables, algorithms, and intended uses (El Arab et al., 2025; Gastaldi et al., 2025; Cozzolino et al., 2025). AI models refined using high-quality local datasets may be especially valuable for improving understanding of local and regional trends, predicting risk more accurately, and informing therapeutic decisions in ways that static conventional models cannot (Goto et al., 2026; Arnold et al., 2025). The findings of this review should also be interpreted in light of important limitations in the underlying evidence base. Much of the literature was retrospective, based on internally derived datasets, and focused primarily on discrimination metrics rather than calibration, clinical utility, workflow consequences, or patient-level outcome. Evidence relating to implementation was comparatively sparse, and few studies directly examined adoption, trust, acceptability, or the organisational requirements necessary for safe integration of AI into routine infection-control practice. The role of AI models within infection-control workflows therefore represents an important area for future research (Radaelli et al., 2024). Once adequately validated, trusted, and integrated into clinical workflows, such models should be deployed in ways that improve efficiency without creating additional workload, ambiguity, or alert burden for clinicians (Tun et al., 2025; Vasey et al., 2022; Samareh et al., 2019). This potential was illustrated by several studies included in this review, in which automation reduced, or could reduce, time spent on selected tasks, including surgical-site-infection surveillance and antimicrobial decision support (Jakobsen et al., 2024b; Cho et al., 2024; Flores-Balado et al., 2023). Yet workflow value is unlikely to depend on model performance alone. It also depends on the availability, quality, and timing of input data; the clinical actionability of model outputs; and the extent to which recommendations can be embedded into existing infection-prevention and antimicrobial-stewardship processes. Pre-admission data and early clinical characteristics may be especially useful for supporting infection-control decisions soon after hospital admission (Zachariah et al., 2020; Jakobsen et al., 2024b). Future studies should therefore evaluate not only statistical performance, but also workflow fit, usability, clinical actionability, staff workload, and safe integration with broader infectious-disease decision-making in routine practice.

A methodological concern is that high discrimination in retrospective datasets may not translate into reliable performance in routine practice. Models developed in single institutions or narrow clinical populations may overfit local coding practices, microbiology workflows, antimicrobial-resistance patterns, surveillance definitions, and electronic-health-record structures. External and temporal validation are therefore particularly important in infection surveillance, where pathogen distribution, testing practices, antimicrobial prescribing, device use, and case definitions vary across hospitals and over time. Calibration is also essential because a model may rank patients accurately while still producing poorly calibrated risk estimates, leading to inappropriate thresholds, excessive alerts, missed cases, or inefficient use of infection-prevention resources (Cozzolino et al., 2025; Vasey et al., 2022). High AUC values should therefore be interpreted cautiously in infection-surveillance and antimicrobial-decision contexts. AUC summarises the ability of a model to rank individuals with and without an outcome across all possible thresholds, but it does not indicate whether predicted risks are well calibrated, whether a selected operating threshold is clinically appropriate, or whether model use improves decisions in practice (Cozzolino et al., 2025; Vasey et al., 2022). In low-prevalence infection outcomes, a model with apparently strong discrimination may still generate many false-positive alerts, increase workload, or produce limited positive predictive value if thresholds are not aligned with clinical priorities and available resources (Cozzolino et al., 2025; Vasey et al., 2022). Conversely, a model with more modest discrimination may be useful if it is well calibrated, embedded at the right point in the workflow, linked to clear action protocols, and shown to reduce manual review, improve antimicrobial targeting, or support timely infection-prevention decisions (Cozzolino et al., 2025; Vasey et al., 2022). In the included literature, studies suggesting operational value, such as reduced manual review for surgical-site-infection surveillance or antimicrobial decision support, remained workflow-proximal rather than definitive evidence of improved patient outcomes (Cho et al., 2024; Bolton et al., 2024; Tsai et al., 2023; Flores-Balado et al., 2023; Kanjilal et al., 2020; Yelin et al., 2019; Oonsivilai et al., 2018). These findings reinforce the need for future studies to report calibration, threshold-selection rationale, decision-curve analysis or other clinical-utility measures, alert burden, comparator performance, and prospective workflow or patient-level effects (Cozzolino et al., 2025; Vasey et al., 2022).

### Implications for practice, research, and policy

For practice, the present synthesis suggests that AI tools relevant to healthcare-associated infections should currently be regarded primarily as potential adjuncts to surveillance, early risk stratification, detection, and selected prescribing-support tasks, rather than as established substitutes for existing infection-control workflows. Their use should therefore remain cautious, context-sensitive, and embedded within clinical oversight, rather than justified on the basis of technical performance alone. For research, the findings indicate a need to move beyond retrospective model-development studies towards stronger prospective, externally validated, and implementation-oriented evaluations. Future studies should assess not only discrimination, but also calibration, clinical utility, workflow consequences, adoption, usability, trust, and patient-level outcomes. Comparative evaluations against existing infection-control pathways, pragmatic implementation studies, and assessments of sustained post-deployment performance are particularly needed. For policy and service governance, the review suggests that adoption decisions should not rely solely on published accuracy metrics. Greater attention is needed to local epidemiology, data quality, antimicrobial-resistance context, workflow fit, accountability, and ongoing performance monitoring. AI tools relevant to healthcare-associated infections should therefore be approached as sociotechnical interventions requiring governance, validation, and implementation planning, rather than as stand-alone algorithmic products.

### Strengths and limitations

This review has several strengths. It addresses an emerging and heterogeneous field using a scoping-review design well suited to mapping the range, nature, and evidentiary maturity of the literature rather than estimating pooled intervention effects. It also distinguished technical performance from clinical utility and implementation relevance *a priori*, thereby reducing the risk of conflating predictive accuracy with demonstrated patient benefit or successful workflow integration. In addition, the review focused specifically on hospital settings, which enhanced contextual coherence in relation to infection-control practice.

This review also has important limitations. First, the included literature was methodologically heterogeneous and therefore required narrative synthesis, limiting direct comparison across studies. Second, much of the evidence base consisted of retrospective studies using internally derived datasets, with relatively limited external validation, prospective evaluation, or direct assessment of implementation outcomes. Third, discrimination-oriented performance metrics were reported far more commonly than calibration, clinical utility, workflow consequences, or patient-level outcomes. Fourth, the review was restricted to English-language publications and to the predefined search period, and no formal protocol was registered. Fifth, although study selection used predefined criteria, reviewer checking, and consensus procedures, fully independent duplicate data extraction and formal risk-of-bias assessment were not conducted across all studies. Finally, a further limitation concerns the potential incompleteness of the evidence base identified by the search strategy. Although multiple major bibliographic databases were searched, complete capture of all relevant studies cannot be guaranteed in a rapidly evolving and inconsistently indexed field. Artificial-intelligence applications relevant to infection surveillance, infection prevention, antimicrobial decision support, and hospital workflow are dispersed across clinical medicine, infection-control, antimicrobial-stewardship, biomedical-informatics, computer-science, and implementation-science literatures. Moreover, relevant studies may not always use the terms “artificial intelligence” or “machine learning” and may instead be indexed or described using terms such as automated surveillance, predictive analytics, electronic surveillance, clinical decision support, statistical learning, digital infection-control systems, or risk modelling. Consequently, some eligible studies may have remained uncaptured. This limitation reinforces the need to interpret the review as a structured scoping map of the identified literature rather than as an exhaustive account of all AI-related infection-control applications.

## Conclusion

AI has meaningful potential to support infection surveillance, risk stratification, infection detection, and selected antimicrobial decision-support tasks in acute-care hospitals. However, the present evidence base remains substantially stronger for retrospective technical performance than for clinical effectiveness, implementation success, or patient benefit. Most studies reported discrimination-oriented metrics, whereas calibration, external and temporal validation, clinical utility, workflow consequences, clinician trust, alert burden, and patient-level outcomes were assessed inconsistently or not at all. AI tools in this field should therefore be regarded as promising adjuncts to infection-prevention and antimicrobial-stewardship practice, not as proven substitutes for established surveillance or clinical decision-making pathways. Future research should prioritise externally validated, prospectively evaluated, workflow-integrated, and implementation-oriented studies that assess whether these systems improve decisions, reduce burden, and benefit patients under routine clinical conditions.

## Data Availability

The original contributions presented in the study are included in the article/Supplementary material, further inquiries can be directed to the corresponding author.
