# Supplementary material for: Artificial intelligence for infection surveillance, risk stratification, and antimicrobial decision support in acute-care hospitals: a scoping review
Source: Front Artif Intell. 2026 Jun 12;9:1859410. doi: 10.3389/frai.2026.1859410 (PMC13303925; doi:10.3389/frai.2026.1859410)
Supplement: Supplementary file 1 [file Data_Sheet_1.docx]

**Supplementary Appendix 1. Full database search strategies**

**Search overview.** The search strategy was developed to identify empirical studies evaluating artificial-intelligence applications relevant to infection surveillance, risk stratification, infection detection, antimicrobial decision support, and workflow-relevant hospital functions in acute-care settings. Searches were conducted in PubMed, Embase, Scopus, Web of Science, CINAHL, and the Cochrane Library. Searches were limited to English-language publications published between Jan 1, 2016, and Jan 31, 2026. Searches were last run on Feb 1, 2026.

The search strategy combined four core concepts: artificial intelligence and related computational methods; infection surveillance, healthcare-associated infections, infection detection, antimicrobial resistance, and antimicrobial decision support; acute-care hospital or inpatient setting; and prediction, surveillance, risk stratification, clinical decision support, implementation, or workflow relevance.

## PubMed

| **Database** | **Search strategy and limits/filters** |
| --- | --- |
| PubMed | ("Artificial Intelligence"[Mesh] OR "Machine Learning"[Mesh] OR "Deep Learning"[Mesh] OR "Natural Language Processing"[Mesh] OR "Clinical Decision Support Systems"[Mesh] OR "artificial intelligence"[tiab] OR AI[tiab] OR "machine learning"[tiab] OR "deep learning"[tiab] OR "neural network*"[tiab] OR algorithm*[tiab] OR "predictive analytic*"[tiab] OR "prediction model*"[tiab] OR "natural language processing"[tiab] OR NLP[tiab] OR "clinical decision support"[tiab] OR "automated surveillance"[tiab] OR "electronic surveillance"[tiab]) AND ("Cross Infection"[Mesh] OR "Infection Control"[Mesh] OR "Surgical Wound Infection"[Mesh] OR "Pneumonia, Ventilator-Associated"[Mesh] OR "Urinary Tract Infections"[Mesh] OR "Bacteremia"[Mesh] OR "Sepsis"[Mesh] OR "Drug Resistance, Microbial"[Mesh] OR "Anti-Bacterial Agents"[Mesh] OR "healthcare-associated infection*"[tiab] OR "health care-associated infection*"[tiab] OR "hospital-acquired infection*"[tiab] OR "hospital acquired infection*"[tiab] OR "nosocomial infection*"[tiab] OR "cross infection*"[tiab] OR "surgical site infection*"[tiab] OR SSI[tiab] OR "ventilator-associated pneumonia"[tiab] OR VAP[tiab] OR "urinary tract infection*"[tiab] OR UTI[tiab] OR bacteremia[tiab] OR bacteraemia[tiab] OR sepsis[tiab] OR "antimicrobial resistance"[tiab] OR "antibiotic resistance"[tiab] OR "antimicrobial stewardship"[tiab] OR "antibiotic prescribing"[tiab] OR "infection surveillance"[tiab] OR "infection prevention"[tiab] OR "infection control"[tiab]) AND ("Hospitals"[Mesh] OR "Hospitalization"[Mesh] OR "Inpatients"[Mesh] OR hospital*[tiab] OR inpatient*[tiab] OR "acute care"[tiab] OR ICU[tiab] OR "intensive care"[tiab] OR "critical care"[tiab]) AND (surveillance[tiab] OR predict*[tiab] OR detect*[tiab] OR diagnos*[tiab] OR "risk stratification"[tiab] OR "risk prediction"[tiab] OR classification[tiab] OR "decision support"[tiab] OR workflow[tiab] OR implementation[tiab] OR usability[tiab] OR adoption[tiab] OR "real-time"[tiab] OR "real time"[tiab]) AND ("2016/01/01"[Date - Publication] : "2026/01/31"[Date - Publication]) AND English[Language]  Limits/filters: Publication date Jan 1, 2016-Jan 31, 2026; English language. |

## Embase

| **Database** | **Search strategy and limits/filters** |
| --- | --- |
| Embase | ('artificial intelligence'/exp OR 'machine learning'/exp OR 'deep learning'/exp OR 'natural language processing'/exp OR 'clinical decision support system'/exp OR 'artificial intelligence':ti,ab OR ai:ti,ab OR 'machine learning':ti,ab OR 'deep learning':ti,ab OR 'neural network*':ti,ab OR algorithm*:ti,ab OR 'predictive analytic*':ti,ab OR 'prediction model*':ti,ab OR 'natural language processing':ti,ab OR nlp:ti,ab OR 'clinical decision support':ti,ab OR 'automated surveillance':ti,ab OR 'electronic surveillance':ti,ab) AND ('hospital infection'/exp OR 'infection control'/exp OR 'surgical wound infection'/exp OR 'ventilator associated pneumonia'/exp OR 'urinary tract infection'/exp OR 'bacteremia'/exp OR 'sepsis'/exp OR 'antimicrobial resistance'/exp OR 'antibiotic agent'/exp OR 'healthcare-associated infection*':ti,ab OR 'health care-associated infection*':ti,ab OR 'hospital-acquired infection*':ti,ab OR 'hospital acquired infection*':ti,ab OR 'nosocomial infection*':ti,ab OR 'cross infection*':ti,ab OR 'surgical site infection*':ti,ab OR ssi:ti,ab OR 'ventilator-associated pneumonia':ti,ab OR vap:ti,ab OR 'urinary tract infection*':ti,ab OR uti:ti,ab OR bacteremia:ti,ab OR bacteraemia:ti,ab OR sepsis:ti,ab OR 'antimicrobial resistance':ti,ab OR 'antibiotic resistance':ti,ab OR 'antimicrobial stewardship':ti,ab OR 'antibiotic prescribing':ti,ab OR 'infection surveillance':ti,ab OR 'infection prevention':ti,ab OR 'infection control':ti,ab) AND ('hospital'/exp OR 'hospital patient'/exp OR 'hospitalization'/exp OR hospital*:ti,ab OR inpatient*:ti,ab OR 'acute care':ti,ab OR icu:ti,ab OR 'intensive care':ti,ab OR 'critical care':ti,ab) AND (surveillance:ti,ab OR predict*:ti,ab OR detect*:ti,ab OR diagnos*:ti,ab OR 'risk stratification':ti,ab OR 'risk prediction':ti,ab OR classification:ti,ab OR 'decision support':ti,ab OR workflow:ti,ab OR implementation:ti,ab OR usability:ti,ab OR adoption:ti,ab OR 'real-time':ti,ab OR 'real time':ti,ab) AND [2016-2026]/py AND [english]/lim  Limits/filters: Publication years 2016-2026; English language. |

## Scopus

| **Database** | **Search strategy and limits/filters** |
| --- | --- |
| Scopus | TITLE-ABS-KEY (("artificial intelligence" OR AI OR "machine learning" OR "deep learning" OR "neural network*" OR algorithm* OR "predictive analytic*" OR "prediction model*" OR "natural language processing" OR NLP OR "clinical decision support" OR "automated surveillance" OR "electronic surveillance") AND ("healthcare-associated infection*" OR "health care-associated infection*" OR "hospital-acquired infection*" OR "hospital acquired infection*" OR "nosocomial infection*" OR "cross infection*" OR "surgical site infection*" OR SSI OR "ventilator-associated pneumonia" OR VAP OR "urinary tract infection*" OR UTI OR bacteremia OR bacteraemia OR sepsis OR "antimicrobial resistance" OR "antibiotic resistance" OR "antimicrobial stewardship" OR "antibiotic prescribing" OR "infection surveillance" OR "infection prevention" OR "infection control") AND (hospital* OR inpatient* OR "acute care" OR ICU OR "intensive care" OR "critical care") AND (surveillance OR predict* OR detect* OR diagnos* OR "risk stratification" OR "risk prediction" OR classification OR "decision support" OR workflow OR implementation OR usability OR adoption OR "real-time" OR "real time")) AND PUBYEAR > 2015 AND PUBYEAR < 2027 AND (LIMIT-TO (LANGUAGE, "English"))  Limits/filters: Publication years 2016-2026; English language. |

## Web of Science Core Collection

| **Database** | **Search strategy and limits/filters** |
| --- | --- |
| Web of Science Core Collection | TS=(("artificial intelligence" OR AI OR "machine learning" OR "deep learning" OR "neural network*" OR algorithm* OR "predictive analytic*" OR "prediction model*" OR "natural language processing" OR NLP OR "clinical decision support" OR "automated surveillance" OR "electronic surveillance") AND ("healthcare-associated infection*" OR "health care-associated infection*" OR "hospital-acquired infection*" OR "hospital acquired infection*" OR "nosocomial infection*" OR "cross infection*" OR "surgical site infection*" OR SSI OR "ventilator-associated pneumonia" OR VAP OR "urinary tract infection*" OR UTI OR bacteremia OR bacteraemia OR sepsis OR "antimicrobial resistance" OR "antibiotic resistance" OR "antimicrobial stewardship" OR "antibiotic prescribing" OR "infection surveillance" OR "infection prevention" OR "infection control") AND (hospital* OR inpatient* OR "acute care" OR ICU OR "intensive care" OR "critical care") AND (surveillance OR predict* OR detect* OR diagnos* OR "risk stratification" OR "risk prediction" OR classification OR "decision support" OR workflow OR implementation OR usability OR adoption OR "real-time" OR "real time"))  Limits/filters: Publication years 2016-2026; English language. |

## CINAHL

| **Database** | **Search strategy and limits/filters** |
| --- | --- |
| CINAHL | (MH "Artificial Intelligence+" OR MH "Machine Learning" OR MH "Decision Support Systems, Clinical" OR TI "artificial intelligence" OR AB "artificial intelligence" OR TI AI OR AB AI OR TI "machine learning" OR AB "machine learning" OR TI "deep learning" OR AB "deep learning" OR TI "neural network*" OR AB "neural network*" OR TI algorithm* OR AB algorithm* OR TI "predictive analytic*" OR AB "predictive analytic*" OR TI "prediction model*" OR AB "prediction model*" OR TI "natural language processing" OR AB "natural language processing" OR TI NLP OR AB NLP OR TI "clinical decision support" OR AB "clinical decision support" OR TI "automated surveillance" OR AB "automated surveillance" OR TI "electronic surveillance" OR AB "electronic surveillance") AND (MH "Cross Infection+" OR MH "Infection Control+" OR MH "Surgical Wound Infection+" OR MH "Pneumonia, Ventilator-Associated" OR MH "Urinary Tract Infections+" OR MH "Bacteremia" OR MH "Sepsis+" OR MH "Drug Resistance, Microbial+" OR MH "Antimicrobial Stewardship" OR TI "healthcare-associated infection*" OR AB "healthcare-associated infection*" OR TI "health care-associated infection*" OR AB "health care-associated infection*" OR TI "hospital-acquired infection*" OR AB "hospital-acquired infection*" OR TI "hospital acquired infection*" OR AB "hospital acquired infection*" OR TI "nosocomial infection*" OR AB "nosocomial infection*" OR TI "cross infection*" OR AB "cross infection*" OR TI "surgical site infection*" OR AB "surgical site infection*" OR TI SSI OR AB SSI OR TI "ventilator-associated pneumonia" OR AB "ventilator-associated pneumonia" OR TI VAP OR AB VAP OR TI "urinary tract infection*" OR AB "urinary tract infection*" OR TI UTI OR AB UTI OR TI bacteremia OR AB bacteremia OR TI bacteraemia OR AB bacteraemia OR TI sepsis OR AB sepsis OR TI "antimicrobial resistance" OR AB "antimicrobial resistance" OR TI "antibiotic resistance" OR AB "antibiotic resistance" OR TI "antimicrobial stewardship" OR AB "antimicrobial stewardship" OR TI "antibiotic prescribing" OR AB "antibiotic prescribing" OR TI "infection surveillance" OR AB "infection surveillance" OR TI "infection prevention" OR AB "infection prevention" OR TI "infection control" OR AB "infection control") AND (MH "Hospitals+" OR MH "Hospitalization+" OR MH "Inpatients+" OR TI hospital* OR AB hospital* OR TI inpatient* OR AB inpatient* OR TI "acute care" OR AB "acute care" OR TI ICU OR AB ICU OR TI "intensive care" OR AB "intensive care" OR TI "critical care" OR AB "critical care") AND (TI surveillance OR AB surveillance OR TI predict* OR AB predict* OR TI detect* OR AB detect* OR TI diagnos* OR AB diagnos* OR TI "risk stratification" OR AB "risk stratification" OR TI "risk prediction" OR AB "risk prediction" OR TI classification OR AB classification OR TI "decision support" OR AB "decision support" OR TI workflow OR AB workflow OR TI implementation OR AB implementation OR TI usability OR AB usability OR TI adoption OR AB adoption OR TI "real-time" OR AB "real-time" OR TI "real time" OR AB "real time")  Limits/filters: Published date 20160101-20260131; English language. |

## Cochrane Library

| **Database** | **Search strategy and limits/filters** |
| --- | --- |
| Cochrane Library | ("artificial intelligence" OR AI OR "machine learning" OR "deep learning" OR "neural network*" OR algorithm* OR "predictive analytic*" OR "prediction model*" OR "natural language processing" OR NLP OR "clinical decision support" OR "automated surveillance" OR "electronic surveillance") AND ("healthcare-associated infection*" OR "health care-associated infection*" OR "hospital-acquired infection*" OR "hospital acquired infection*" OR "nosocomial infection*" OR "cross infection*" OR "surgical site infection*" OR SSI OR "ventilator-associated pneumonia" OR VAP OR "urinary tract infection*" OR UTI OR bacteremia OR bacteraemia OR sepsis OR "antimicrobial resistance" OR "antibiotic resistance" OR "antimicrobial stewardship" OR "antibiotic prescribing" OR "infection surveillance" OR "infection prevention" OR "infection control") AND (hospital* OR inpatient* OR "acute care" OR ICU OR "intensive care" OR "critical care") AND (surveillance OR predict* OR detect* OR diagnos* OR "risk stratification" OR "risk prediction" OR classification OR "decision support" OR workflow OR implementation OR usability OR adoption OR "real-time" OR "real time")  Limits/filters: Publication date Jan 1, 2016-Jan 31, 2026; English language. |

**Note.** Search strings were adapted to the indexing structure and syntax of each database. Controlled vocabulary terms were used where available and supplemented with free-text terms searched in title and abstract fields or equivalent topic fields. Retrieved records were exported to reference-management software for deduplication before title and abstract screening.
